# Acid-induced demulsification *versus* alum and polyaluminium chloride coagulation for oil and turbidity removal from concentrated soybean oil-in-water emulsions

**DOI:** 10.1039/d6ra05919h

**Published:** 2026-10-21

**Authors:** Praveen Kumar, Shilpi Verma, Amit Verma, Vimal Chandra Srivastava

**Affiliations:** a Faculty of Chemistry and Chemical Technology, University of Ljubljana 1000 Ljubljana Slovenia praveen@engineering.ucsb.edu Kumar.Praveen@fkkt.uni-lj.si; b Department of Energy & Environment, Thapar Institute of Engineering & Technology Patiala-147004 Punjab India; c Department of Chemical Engineering, Indian Institute of Technology Roorkee 247667 India

## Abstract

This study evaluated the treatment of concentrated soybean oil-in-water emulsions by acid-induced demulsification using hydrochloric acid (HCl) and by coagulation with polyaluminum chloride (PAC) and alum. The impact of chemical dose, pH, and settling time was examined to determine the effectiveness of each process in oil and turbidity removal. Acid treatment reduced turbidity by 97.1% through phase separation at low pH but was not sufficient to bring residual oil below discharge standards. PAC and alum both demonstrated strong pH- and dose-dependent removal behaviors, with optimal performance at pH 7 for PAC and pH 6 for alum. Among the tested systems, the maximum removal of oil and turbidity was observed using optimized alum coagulation in a two-stage process, achieving oil removal up to 82% and turbidity removal of over 96%, which outperformed HCl and PAC, especially for high-strength emulsions. These findings identify alum-based systems as the most effective conventional choice for high-percentage oil and turbidity removal in concentrated soybean oil emulsions.

## Abstract 2

This study evaluated the treatment of concentrated soybean oil-in-water emulsions through acid-induced demulsification using hydrochloric acid (HCl) and coagulation using polyaluminum chloride (PAC) and alum. The image was created with the assistance of ChatGPT Business (OpenAI).

## Graphical abstract

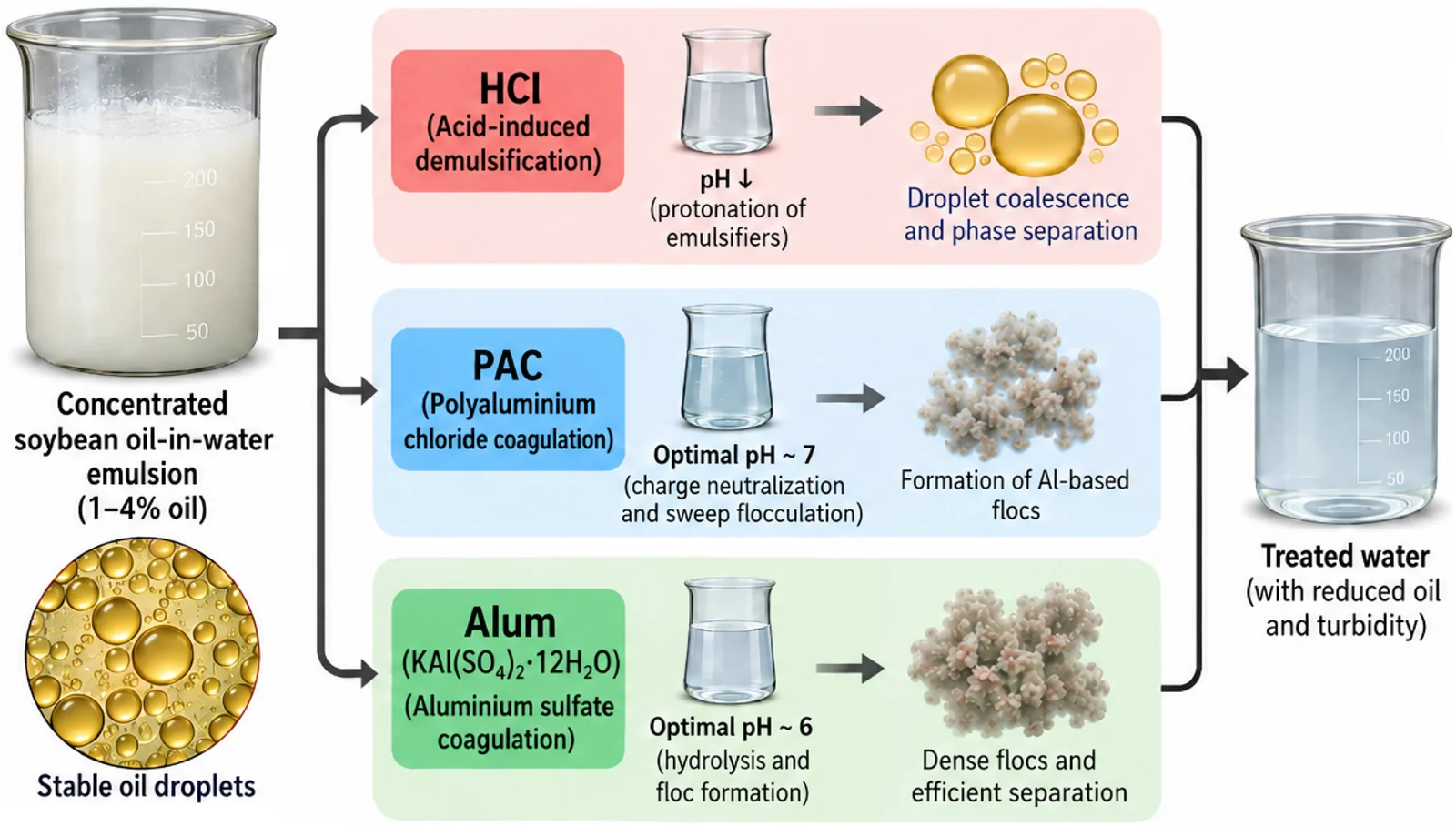

## 1. Introduction

Global growth in petroleum refining, petrochemical production, metal-working, vegetable-oil manufacturing, automotive assembly, and dairy or food processing industries has markedly increased the discharge of oil- and grease-rich wastewaters (O&G). Typical O&G concentrations in refinery desalter effluents, metal-cutting fluids, or produced waters, range from 1000–30 000 mg L^−1^, while the chemical oxygen demand (COD) frequently exceeds 10 g L^−1^, salinity ranges from 17–23 g L^−1^, and total dissolved solids exceed 26 g L^−1^. When inadequately treated, these effluents form surface films that inhibit oxygen transfer, impair photosynthesis, foul the sewer networks, and introduce toxic polycyclic aromatic hydrocarbons and heavy metals into aquatic ecosystems.^1^ Recognizing these risks, the environmental agencies, throughout the world, now prescribe stringent discharge limits. The Central Pollution Control Board in India maintains a discharge limit of 10 mg L^−1^ O&G for inland surface-water release,^2^ and the Environmental Protection Agency (EPA) in the United States allows ≤35 mg L^−1^ for produced water to be used in livestock or irrigation.^3^ Achieving such ultra-low targets is challenging because industrial streams often contain highly stable oil-in-water (o/w) emulsions composed of droplets smaller than 3 µm that remain dispersed by surfactants, polymers, fine solids, and extreme salinity.^4^

Conventional gravity settlers, API separators, and induced-gas or dissolved-air flotation units remove free oil but are not effective in reducing the residual O&G below 50–100 mg L^−1^.^5^ Chemical coagulation–flocculation (C/F) with alum, ferric salts or polyaluminium chloride (PAC), therefore provides an effective solution for breaking the emulsion because it can operate across wide pH and salinity ranges, provides fast destabilisation, and is easy to retrofit.^6,7^ Within this technological landscape, vegetable-oil emulsions—particularly soybean oil systems—serve as model substrates for fundamental demulsification research because they emulate droplet size distributions and surface chemistries found in food, metal-working, and agro-processing effluents. Soybean oil contamination in water originates from multiple industrial and commercial sources, ranging from large-scale manufacturing to small-scale food service operations, each contributing distinct characteristics and treatment challenges. During soybean oil extraction and refining processes, operations such as degumming, neutralization, bleaching, and deodorisation generate effluents with complex compositions including phospholipids, free fatty acids, surfactants, and residual processing chemicals.^8^ The refined soybean oil industry generates substantial wastewater containing high concentrations of oils, organic matter, and processing chemicals. The presence of phospholipids in soybean processing wastewater tends to contribute to higher biomass content and enhances microbial lipid production when subjected to biological treatment. Refined soybean oil wastewater (RSOW) typically exhibits chemical oxygen demand (COD) values between 32 600 and 86 700 mg L^−1^.^8,9^ Soybean oil serves as a primary feedstock for biodiesel production on an industrial scale, with first-generation biodiesel commonly produced from virgin vegetable oils including soybean, rapeseed, sunflower, palm, and coconut oil.^10–12^ The biodiesel purification process generates extremely high-strength wastewater with COD values reaching 667 000 mg O_2_ per L making it one of the most challenging industrial wastewaters to treat.^13^ Food manufacturing operations using soybean oil as an ingredient also contribute to oily wastewater generation. Processing plants manufacturing soybean-based products discharge effluents with varying oil concentrations depending on specific operations, including oil extraction, protein isolation, and product formulation.^14^ The alkaline extraction methods commonly used in protein processing generate large amounts of wastewater that can lead to environmental hazards if improperly managed.^15^ Restaurant kitchens and food service establishments represent significant point sources of soybean oil contamination in municipal wastewater systems. Restaurant wastewater typically contains O&G concentrations up to 3434 mg L^−1^, substantially exceeding municipal treatment plant design parameters.^16,17^ Automotive manufacturing and parts cleaning operations may utilize soybean-based degreasers and cleaning agents, contributing to industrial wastewater streams containing vegetable oil emulsions. The shift toward bio-based industrial fluids has increased the prevalence of soybean oil derivatives in various manufacturing wastewaters. Soybean meal production and livestock feed manufacturing generate wastewater containing both oil residues and protein-rich organics.^18^ These operations create complex wastewater matrices that require specialized treatment approaches due to the combination of lipids, proteins, and other organic compounds. Used cooking oil (UCO) collection and processing operations handling soybean-based oils generate secondary waste streams during recovery operations. Cooking oil secondary waste (COSW) typically contains 53% oil content, creating highly concentrated waste streams requiring intensive treatment.^19^ These facilities process waste oils from restaurants, food manufacturers, and households, creating concentrated oily waste streams during separation and purification operations. The diversity of soybean oil sources in wastewater creates complex treatment challenges due to varying oil concentrations, emulsion stability, and co-contaminant interactions.^16^ The coagulation process is a commonly employed technique in both water and wastewater treatment due to its effectiveness in destabilizing and aggregating colloidal particles. A study was done by Bou *et al.*^20^ on the application of chemical coagulation for the removal of oil from industrial wastewater and the effect of initial pH, coagulant type, and dosage was investigated for the separation efficiency at various oil concentrations. Aluminum sulphate at 800 mg L^−1^ removed up to 80% of oil at 1–2 g L^−1^ and 90% at 3 g L^−1^ (pH 7–8). Ferric chloride, applied at 500–900 mg L^−1^, achieved up to 90% oil removal across all tested concentrations. At 700 mg L^−1^, ferric chloride outperformed aluminum sulphate, which removed only 60–85%. Song *et al.*^21^ proposed a novel demulsification strategy using PAC for charge neutralization, followed by a demulsifier to improve oil–water separation. They optimized temperature and electric field settings for the dehydration process. PAC effectively reduced electronegativity, improved electric field response, and increased dehydration rates. Ahmad *et al.*^22^ compared the effectiveness of alum, polyaluminum chloride (PAC), and biodegradable chitosan as coagulants in treating palm oil mill effluent (POME). Under optimal conditions (0.5 g L^−1^ dosage, 15 min contact, 100 rpm mixing, 20 min sedimentation, pH 4), chitosan removed over 95% of suspended solids and residual oil. In contrast, alum and PAC required higher dosages (8.0 and 6.0 g L^−1^), longer contact and sedimentation times, and a slightly higher pH (4.5) to achieve the same removal efficiency.

While prior studies have examined coagulation of oily wastewaters using alum, PAC, or chitosan individually, or have focused on palm oil or produced-water systems,^20–22^ few have directly compared acid-based demulsification with conventional metal-salt coagulants under a controlled, standardized emulsion matrix, and fewer still have evaluated whether single- or multi-stage treatment can meet stringent discharge limits below 10 mg L^−1^. Concentrated HCl, in particular, has been applied for crude oil demulsification through interfacial film protonation and charge neutralization,^25–27^ but its performance on protein- and phospholipid-stabilized vegetable oil emulsions, such as soybean oil, remains largely unexplored. This study addresses this gap by systematically comparing HCl, PAC, and alum across a common range of soybean oil concentrations (1–4% v/v), evaluating dose, pH, and retention-time dependence, and determining the minimum treatment stages required to reach regulatory compliance, thereby providing a direct mechanistic and practical benchmark among acid-, polymer-, and hydrolysis-based demulsification strategies for this class of emulsion.

## 2. Materials and methods

### 2.1 Materials

The polyaluminium chloride (PAC) used was a commercial liquid formulation, Vikram PAC Liquid (Grade SAB18), procured from Grasim Industries Ltd (India), with the following specifications as supplied by the manufacturer: [Al_2_O_3_ content: 16.5–17.5% (w/w); basicity: 35%; specific gravity (25 °C): 1.35–1.39; appearance: pale yellow to amber clear liquid]. Alum (KAl(SO_4_)_2_·12H_2_O), sulfuric acid (H_2_SO_4_), sodium hydroxide (NaOH), and hydrochloric acid (HCl) were of analytical grade and were purchased from LOBA Chemie Pvt. Ltd (Mumbai, India). All chemicals were used as received without further purification, and all solutions were prepared using distilled water.

### 2.2 Equipment setup and methodology

Soybean oil was used to make the synthetic oil–water emulsion. Oil–water emulsions of different oil concentrations were made by sonication with distilled water. The Misonix XL 2020 ultrasound processor, equipped with a horn tip of 1.27 cm in diameter, was used to generate a 20 kHz ultrasound for creating oil–water emulsions. Oil–water emulsions with oil concentrations of around 1%, 2%, 3%, and 4% (v/v) with respective oil concentrations of 9020; 18 040; 27 060 and 36 080 mg L^−1^, were prepared and used in this study. At these oil concentrations, the respective turbidities were 10 000; 20 000; 30 000 and 40 000 NTU. The oil–water mixture was subjected to ultrasonic treatment using the Misonix XL 2020 processor at an intensity of 126 W cm^−2^ and fixed frequency of 20 kHz. Sonication was continued until a uniform milky-white appearance was obtained, indicating the formation of a stable oil-in-water emulsion. This visual observation was used as the endpoint for emulsion preparation before subsequent coagulation studies.

Coagulation–flocculation experiments followed the standardized jar test procedure at 25 °C.^23^ The baseline turbidity measurements were recorded using a nephelometric turbidimeter. Initial pH values were systematically adjusted to predetermined levels using 0.05 N HCl or 0.05 N NaOH solutions to evaluate pH-dependent coagulation efficiency. The predetermined quantities of coagulants (alum and PAC) were added to individual beakers containing the emulsion. The three-stage mixing protocol comprised: (1) rapid flash mixing at high magnetic stirrer speed for 1 minute at 150 rpm to ensure thorough coagulant distribution throughout the aqueous phase; (2) gentle flocculation mixing for 15 minutes at 20 rpm to promote floc formation and growth; and (3) quiescent settling for 30 minutes to allow gravitational separation of coagulated oil particles. Supernatant samples were carefully collected from a standardized depth of 2 cm below the surface to avoid disturbing settled flocs. Analytical parameters included pH, residual turbidity, and oil concentration measurements. The experimental conditions and treatment outcomes for all soybean oil concentrations are summarized in Table S1. Time-series experiments monitored turbidity reduction in the supernatant at predetermined intervals to establish optimal retention times for each coagulant system. Residual oil concentrations in supernatant samples were quantified using the petroleum ether extraction method prescribed by Bureau of Indian Standards.^24^ Samples exceeding the target discharge limit of 10 mg L^−1^ were subjected to secondary treatment using identical coagulant systems. The experimental runs for both the single- and two-stage configurations were repeated in triplicate using independently prepared emulsion batches. The two-stage approach involved re-treating the primary supernatant with optimized coagulant dosages until residual oil concentrations met regulatory discharge standards. This sequential treatment strategy simulated real-world applications requiring multi-stage processes for challenging wastewater matrices. Experiments involving concentrated HCl for de-emulsification were performed following the procedures outlined by Silin *et al.*^25^ Results are presented as mean values, with error bars representing ±5% of the corresponding mean.

## 3. Results and discussion

### 3.1 Treatment with acid: acid-induced demulsification

The treatment of concentrated soybean oil–water emulsions using hydrochloric acid (HCl) as a demulsifying agent demonstrates remarkable effectiveness in achieving turbidity reduction through a distinct biphasic mechanism.

#### 3.1.1 Effect of concentrated HCl dose

Fig. 1 shows the effect of HCl dose on a 1 to 4% oil–water emulsion to obtain minimum turbidity and oil concentration. The relationship between oil concentration and acid dose requirements in oil–water emulsion systems follows a complex, non-proportional pattern governed by colloidal stability principles. As demonstrated in soybean oil–water emulsions ranging from 1% to 4% concentration, the HCl dosage requirement exhibits nonlinear behavior that deviates significantly from simple stoichiometric relationships.^26,27^ This phenomenon can be explained through the Derjaguin–Landau–Verwey–Overbeek (DLVO) theory, which describes the balance between attractive van der Waals forces and electrostatic repulsion in colloidal systems.^28^ The interfacial tension dynamics play an important role in determining emulsion stability and subsequent acid requirements.^29^ Research demonstrates that interfacial tension between oil and water phases directly influences droplet coalescence mechanisms and the effectiveness of chemical destabilization agents. The increased interfacial area requires proportionally greater acid dosing at higher oil concentrations to achieve sufficient electrostatic charge neutralization and interfacial film disruption.^21,31^

**Fig. 1:**
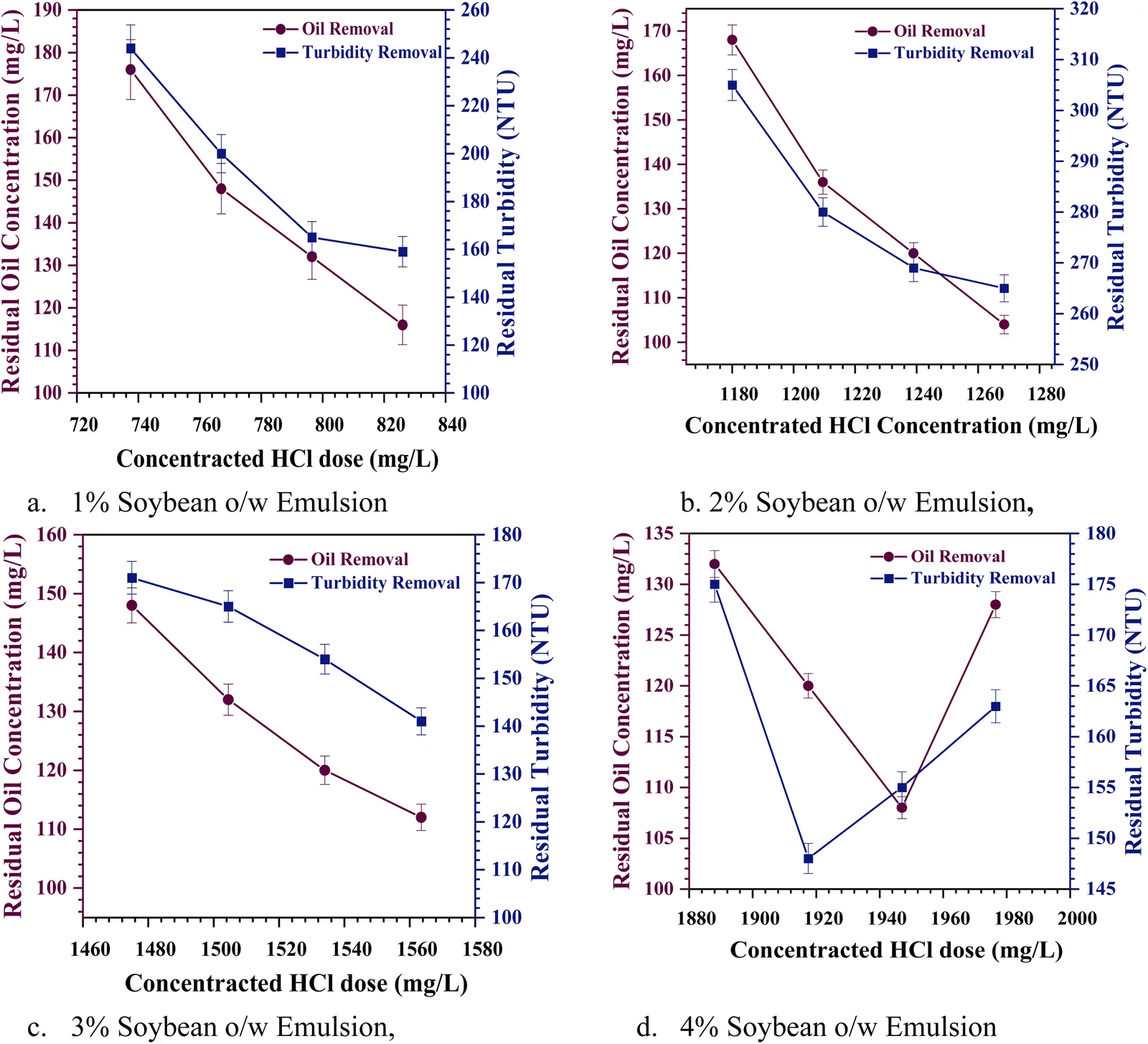
Effect of concentrated HCl dosage on residual oil concentraction and turbidity in soybean oil–water emulsions containing (a) 1%, (b) 2%, (c) 3% and (d) 4% soybean oil.

The concentrated HCl treatment induces emulsion destabilization through multiple concurrent mechanisms. Primary destabilization occurs *via* protonation of natural emulsifying agents present at the oil–water interface, including naphthenic acids and asphaltene-type compounds.^27,31^ Secondary mechanisms involve the modification of interfacial rheological properties through acid-induced changes in interfacial viscosity and elasticity.^30^ The Marangoni effect becomes significant at higher acid concentrations, where interfacial tension gradients drive spontaneous emulsification or destabilization depending on system conditions.^32^ The net effect depends strongly on local conditions, such as acid concentration, interfacial composition, and mixing regime. Under certain circumstances, particularly where strong gradients and turbulence are present, localized emulsification may briefly occur and generate smaller droplets. However, in the context of this study, the applied concentrated HCl and the optimized process parameters result predominantly in destabilization, where interfacial tension gradients promote droplet coalescence and phase separation rather than sustained fine droplet creation. Thus, despite the potential for local spontaneous emulsification, the overall observed result at optimized acid doses is effective demulsification and oil–water separation. This distinction helps interpret the role of Marangoni effects and underscores the importance of controlled dosing and mixing in achieving reliable demulsification performance.^33,34^

However, even at the optimum acid dose—corresponding to the lowest measured turbidity—the residual oil content in the aqueous phase remained above the permissible discharge limit of 10 mg L^−1^. The residual oil concentration for 1% to 4% soybean o/w emulsion varied from 115 mg L^−1^ to 128 mg L^−1^, as shown in Fig. 1. To attempt further removal, the separated supernatant was subjected to a second stage of acid treatment. This secondary treatment did not significantly reduce oil concentration, indicating that the remaining oil exists predominantly as stable submicron droplets or emulsified microphases that are not susceptible to further destabilization *via* acid addition.^35^ The findings thus indicate that concentrated HCl is effective in breaking high-concentration oil–water emulsions, where the interfacial films are abundant and aggressively disrupted by the acid.^36^ In addition to the established mechanism of interfacial film protonation by hydrochloric acid, it is important to consider the role of complex interfacial structures in soybean oil emulsions. The oil–water interface in these systems is often stabilized by proteins, phospholipids, and their associated complexes, which contribute to both the stability and breakdown pathways of the emulsion. Acidification not only protonates amino and carboxyl groups on interfacial proteins, leading to loss of surface charge and film integrity, but may also disrupt protein–lipid interactions and promote hydrolysis or saponification of phospholipids—processes known to contribute to the destabilization and demulsification of soy and other vegetable oil emulsions. The combined effects of these mechanisms under acid treatment help explain the observed efficient breakdown of emulsion structures in this study and are in alignment with mechanistic insights from related work on enzymatic and acid-induced demulsification of oil–protein emulsions.^37,38^

In contrast, once the residual oil concentration is low, the remaining droplets are stabilized by strong interfacial films or micellar/colloidal structures, and the system approaches a thermodynamic and kinetic limit.^34^

#### 3.1.2 Effect of settling time

The experimental results, showing a reduction from an initial turbidity of 10 000 NTU to a final value of 290 NTU (97.1% reduction) over an 8 hour treatment period, are shown in Fig. 2. The settling process exhibits two distinct phases: a rapid initial phase achieving 96.8% turbidity removal in the first 4 hours, followed by a slower secondary phase contributing an additional 0.3% removal efficiency. The rapid initial phase demonstrates the effectiveness of hydrochloric acid in destabilizing the oil–water emulsion through pH reduction and charge neutralization mechanisms. Research has established that acid coagulation operates primarily through the reduction of zeta potential values of oil droplets, leading to weakened electrostatic repulsion and enhanced aggregation potential.^39^ During the initial rapid phase (0–4 hours), hydrochloric acid acts as a destabilizing agent by reducing pH and decreasing the zeta potential of oil droplets, leading to weakened electrostatic repulsion and coalescence of emulsified droplets. This chemical action promotes the aggregation and flocculation of oil particles into larger, more buoyant structures. Critically, the subsequent removal of these aggregates from the aqueous phase is governed by density-driven flotation, not gravitational sedimentation. Because oil has a lower density than water, the coalesced oil droplets and oil-rich flocs naturally migrate upward toward the surface rather than settling downward. This upward separation is fundamentally different from the gravity-driven sedimentation of mineral particles or dense suspended solids, which occur in conventional treatment systems. Thus, the observed turbidity reduction (from 10 000 NTU to 290 NTU over 8 hours) reflects the combined effect of acid-catalyzed coalescence followed by buoyancy-driven phase separation. Clarifying this mechanistic distinction is important for understanding the treatment dynamics and differentiating oil–water emulsion separation from conventional particle sedimentation processes.^40,41^

**Fig. 2:**
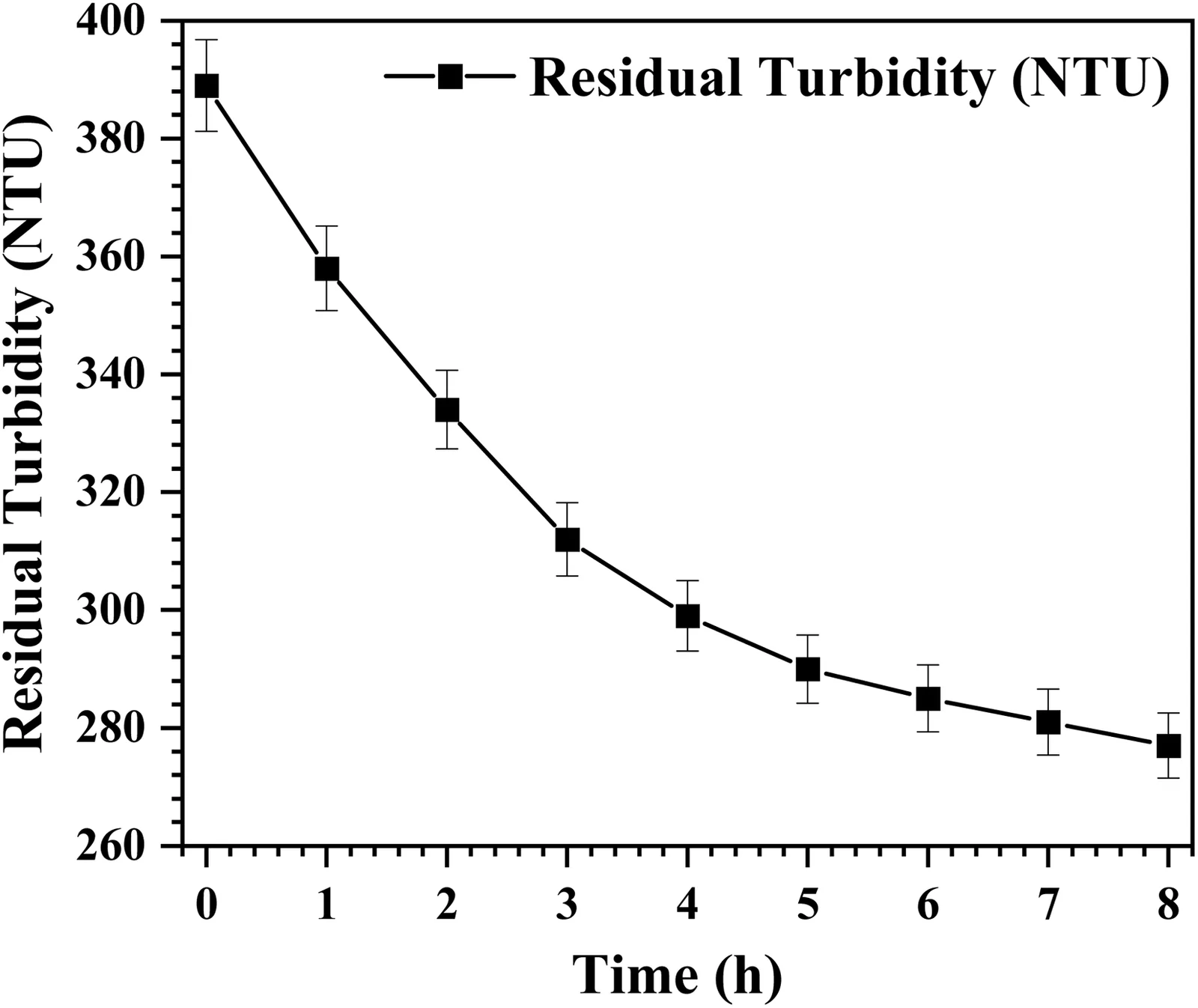
Residual turbidity *vs.* time for the 1% soyabean o/w emulsion using concentrated HCl as a demulsifying agent (initial oil concentration: 9020 mg L^−1^; initial turbidity: 10 000 NTU; concentrated HCl dose: 737.5 mg L^−1^).

A study done by Lamanna *et al.*^42^ on oil–water emulsion destabilization indicates that acid treatment can effectively disrupt protein-stabilized interfaces common in vegetable oil systems, leading to enhanced coalescence and separation efficiency.^44^

### 3.2 Treatment with PAC

PAC as a coagulant has been developed and used in water and wastewater treatment throughout the world. PAC can rapidly produce large and dense flocs at low dosages, resulting in efficient demulsification and reduced oil content in the treated effluent, often outperforming traditional coagulants.^43^

#### 3.2.1 Effect of pH

Fig. 3 demonstrates the pH-dependent coagulation efficiency of PAC in treating soybean oil emulsions, revealing the critical relationship between solution chemistry and treatment performance. The results show that oil and turbidity removal efficiencies increase with pH up to an optimum of 7.0, beyond which both efficiencies decrease, as shown in Fig. 3. For the oil–water emulsion having an initial oil concentration of 1% (v/v *i.e.* 9020 mg L^−1^), PAC dosage of 1200 mg L^−1^, maximum turbidity and oil removal were found as 91.60% when the pH was 7 (Fig. 3). At low pH conditions (pH < 5), turbidity remains elevated due to the predominance of mononuclear aluminum species (Al_3_^+^, Al(OH)^2+^) that provide limited bridging capacity for oil droplet aggregation. The insufficient hydrolysis of PAC at acidic conditions results in reduced coagulation efficiency as the aluminum species lack the optimal charge density and size distribution required for effective destabilization.^45^ Within the optimal pH range (6.0–7.5), turbidity reaches its minimum value of 8–10 NTU, corresponding to maximum coagulation efficiency. This pH zone represents the optimal speciation of medium polymeric aluminum species (Al_13_, Al_30_) that exhibit superior charge neutralization and bridging capabilities. The Al_13_ species, characterized by its Keggin structure [Al_13_O_4_(OH)_24_(H_2_O)_12_]^7+^, demonstrates exceptional surface complexation properties with negatively charged oil droplets. Simultaneously, the larger Al_30_ species provides enhanced inter-particle bridging through its extended polymer structure, facilitating sweep flocculation mechanisms.^46,47^ At higher pH conditions (pH > 8), turbidity increases progressively due to aluminum hydroxide precipitation and charge reversal phenomena. The formation of Al(OH)_3_ precipitates reduces the availability of active coagulant species while potentially restabilizing oil droplets through steric stabilization mechanisms. Additionally, charge reversal may occur where excess aluminum hydroxide species impart positive charges to oil droplet surfaces, creating electrostatic repulsion that opposes aggregation.^45,48,49^ The Henderson–Hasselbalch relationships controlling these equilibria dictate that slight pH variations can significantly alter the distribution of aluminum species, directly impacting coagulation performance. This pH dependence necessitates precise pH control in practical applications to maintain optimal treatment efficiency and consistent performance in oil–water emulsion treatment systems.^50,51^

**Fig. 3:**
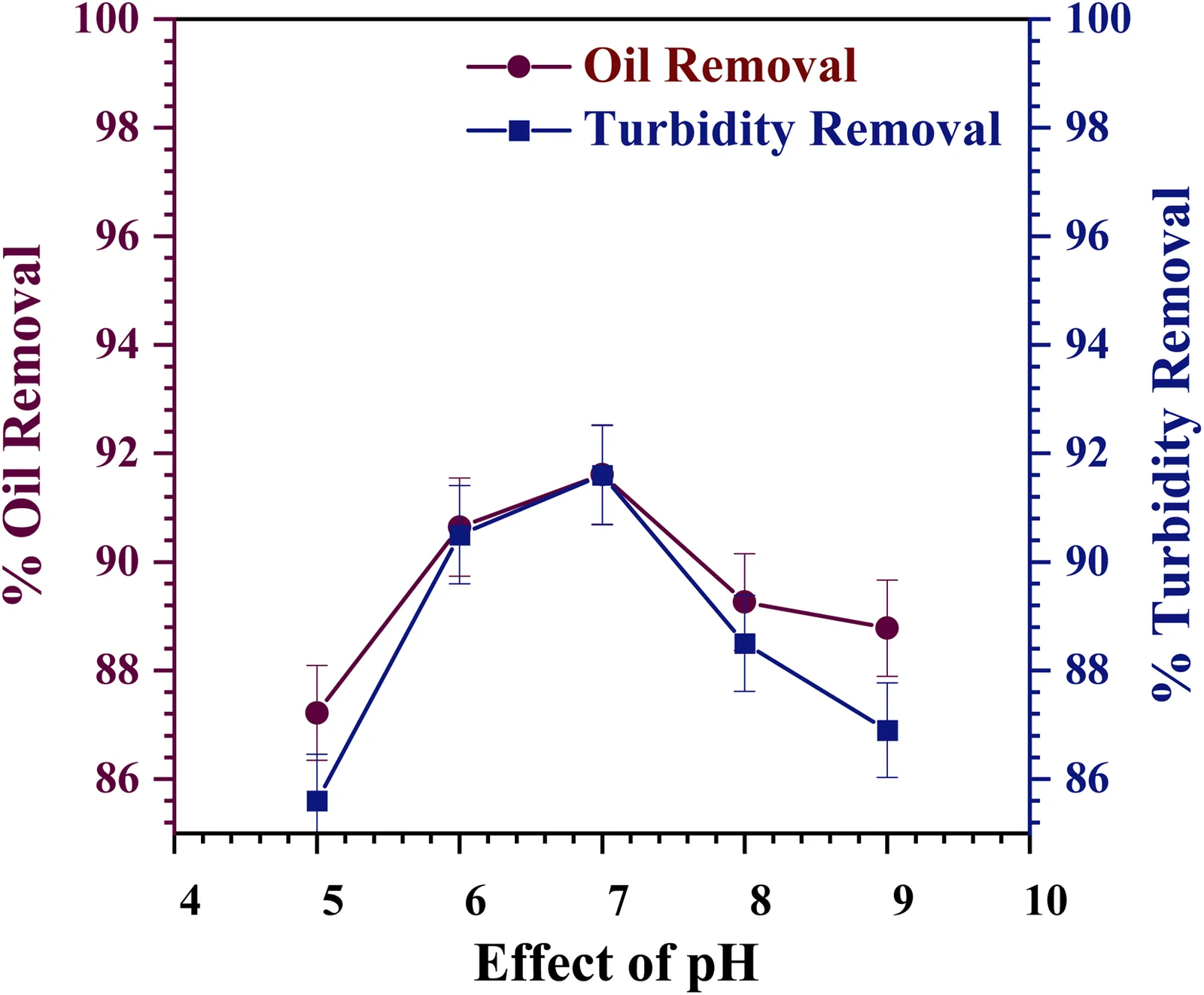
Residual turbidity *vs.* pH of 1% soybean o/w emulsion using PAC as a coagulant (initial oil concentration: 9020 mg L^−1^; initial turbidity: 10 000 NTU; PAC dose: 1200 mg L^−1^).

#### 3.2.2 Effect of PAC dosages

Different amounts of PAC were used with emulsions of different oil concentrations to determine the optimum dosage, as shown in Fig. 4a–d. For 1% o/w emulsion, the PAC dosage of 1300 mg L^−1^, resulted in minimum residual oil and turbidity of 652 and 743 mg L^−1^, respectively. Similarly, for 2%, 3% and 4% o/w emulsion, the minimum residual oil and turbidity was achieved with PAC dosage of 1700, 2150, and 2350 mg L^−1^. The results revealed the U-shaped trend in residual oil concentration and turbidity for the 1% and 2% emulsions, with both parameters decreasing as PAC dosage increased to an optimum, followed by an increase at higher dosages. For the 3% and 4% emulsions, residual oil concentration and turbidity generally decreased over the investigated dosage range (Fig. 4(a–d)). This phenomenon correlates with the fundamental coagulation mechanisms involving charge neutralization and sweep flocculation, where the optimal dosage represents the point of maximum destabilization of the emulsified oil droplets.^51^ Unlike simple colloidal systems, soybean oil droplets are stabilized by a complex interfacial matrix composed primarily of proteins and phospholipids, which impart unique electrostatic and steric characteristics to the emulsion. These bio-macromolecules at the oil–water interface can alter both the adsorption dynamics of PAC-derived Al species and the local hydrolysis equilibrium, sometimes leading to distinctive floc morphologies and responsiveness to optimal and excess PAC dosing. Interfacial proteins and phospholipids may hinder or facilitate charge neutralization and floc formation, and can also affect the size and density of residual flocs, which is not typically observed in conventional mineral colloid suspensions. Thus, the observed persistence of stable oil droplets even after optimized or secondary PAC treatment is in part a reflection of these soybean-derived interfacial structures, which must be considered in modeling and optimizing coagulation protocols for oily effluents.^52,53^

**Fig. 4:**
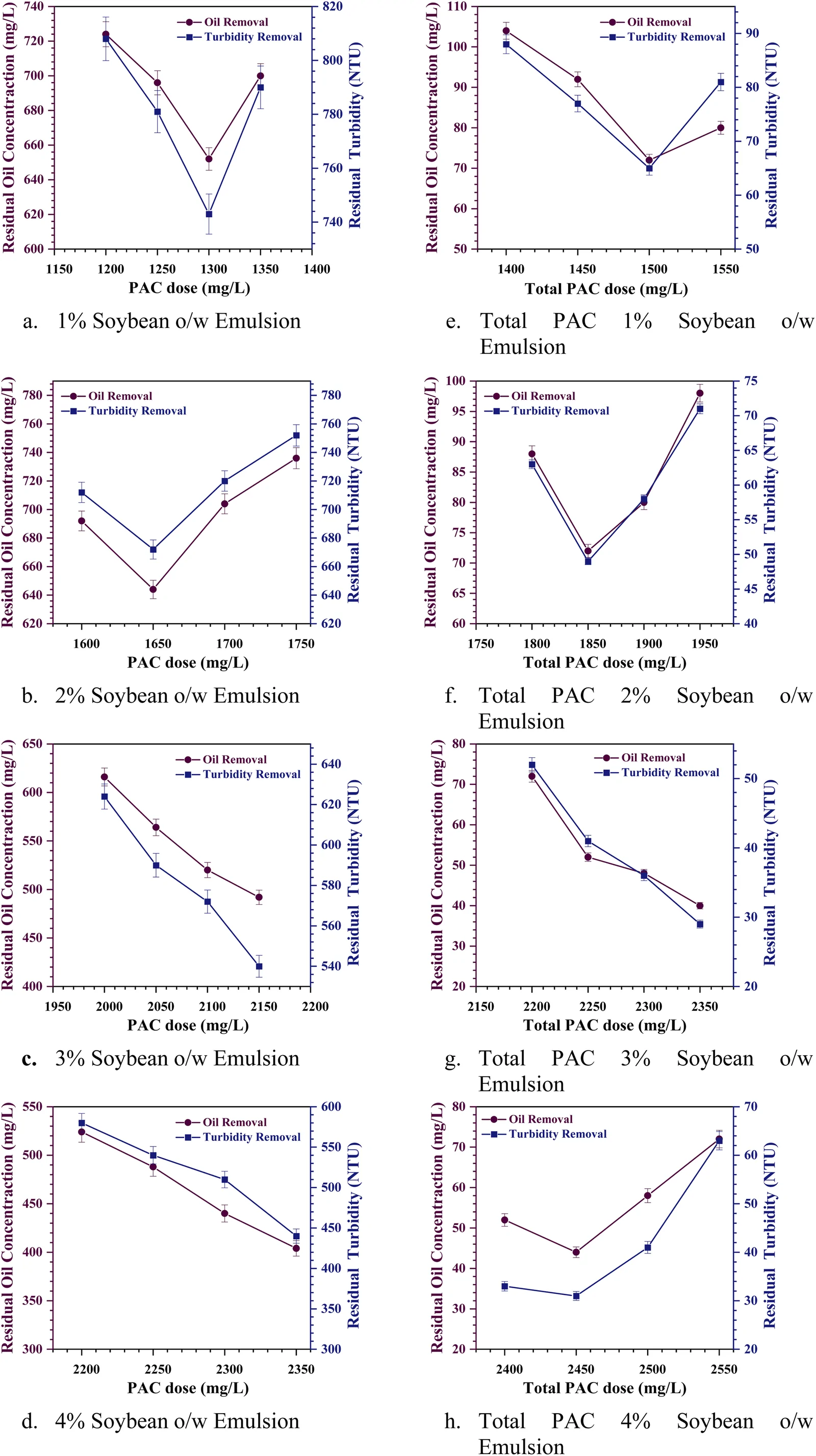
Effect of PAC dosage on residual oil concentration and turbidity of soybean oil–water emulsions. (a–d) First-stage coagulation of 1%, 2%, 3%, and 4% soybean oil-in-water emulsions, respectively. (e–h) Second-stage coagulation of the supernatants obtained after the respective first-stage treatments. A fixed additional PAC dose of 200 mg L^−1^ was applied during the second-stage treatment. The PAC dosages displayed in panels (e–h) represent the cumulative PAC dose, calculated as the first-stage PAC dose plus 200 mg L^−1^.

The observed decrease in removal efficiency beyond the optimal dose is consistent with the re-stabilization phenomenon extensively documented in coagulation literature.^51,54^

Despite optimization of the coagulation process, the single-stage treatment failed to achieve the target effluent oil concentration of 10 mg L^−1^ across all tested emulsion concentrations. The residual oil content in the supernatant following primary treatment consistently exceeded regulatory discharge limits, necessitating the implementation of a two-stage coagulation–flocculation system.^54^ The PAC dosages used in the two-stage treatment are listed in Table S2. The implementation of the secondary treatment stage was designed to address the limitations of single-stage processing in achieving stringent oil removal requirements. However, experimental results demonstrated that even after the second-stage treatment, the oil content remained above the specified limit of 10 mg L^−1^, as shown in Fig. 4e–h. This performance limitation can be attributed to several factors,^20,55^ such as higher oil concentrations create more stable emulsions that are increasingly resistant to destabilization, fine oil droplets that escape primary coagulation require enhanced treatment approaches and at higher oil loadings, the coagulation kinetics become limited by the availability of coagulant species relative to the oil droplet surface area.^56,57^ In addition, in soybean oil emulsions, the proteins and phospholipids at the oil–water interface create robust, nanometric-scale colloidal domains (often <100 nm) that are thermodynamically stable or kinetically trapped. These nanostructures resist conventional coagulation–flocculation due to their extremely small size, high surface-area-to-volume ratios, and strong interfacial stabilization by biomolecules. Additionally, the formation of microemulsified domains—where surfactants and natural emulsifiers form closed-shell structures around oil cores—further impedes aggregation and removal through simple destabilization mechanisms.^58^

#### 3.2.3 Effect of settling time

Having optimized the pH and dose, time-dependent clarification behavior was examined next to understand floc growth and separation kinetics. To study the effect of settling time on the demulsification process, a dose of 1200 mg L^−1^ of PAC was added to the emulsion at an optimum pH of 7 (Fig. 5). The initial oil concentration in the emulsion was 1% (9020 mg L^−1^). The experimental observations revealed phases characteristic of oil–water emulsion treatment by coagulation–flocculation. During the initial phase (0–90 minutes), the rate of floc formation was relatively slow, attributed to the stabilized nature of the emulsion droplets and the time required for charge neutralization and polymer bridging mechanisms to destabilize the oil droplets. The formed flocs tended to migrate upward, creating a thin layer of flocculated material at the surface, consistent with the lower density of oil compared to water and the buoyancy-driven separation typical in oil–water systems.^42^ The turbidity removal kinetics exhibited a biphasic behavior with a notable transition at approximately 90 minutes of retention time. Beyond this critical point, the rate of residual turbidity reduction became significantly slower. It is primarily governed by the migration of buoyant, oil-rich flocs upward (flotation) rather than by classic gravitational settling. This is due to the lower density of oil compared to water, so the dominant mechanism of phase separation is flotation rather than sedimentation.^59^ By the end of 8 h, the residual oil concentration was 850 mg L^−1^.

**Fig. 5:**
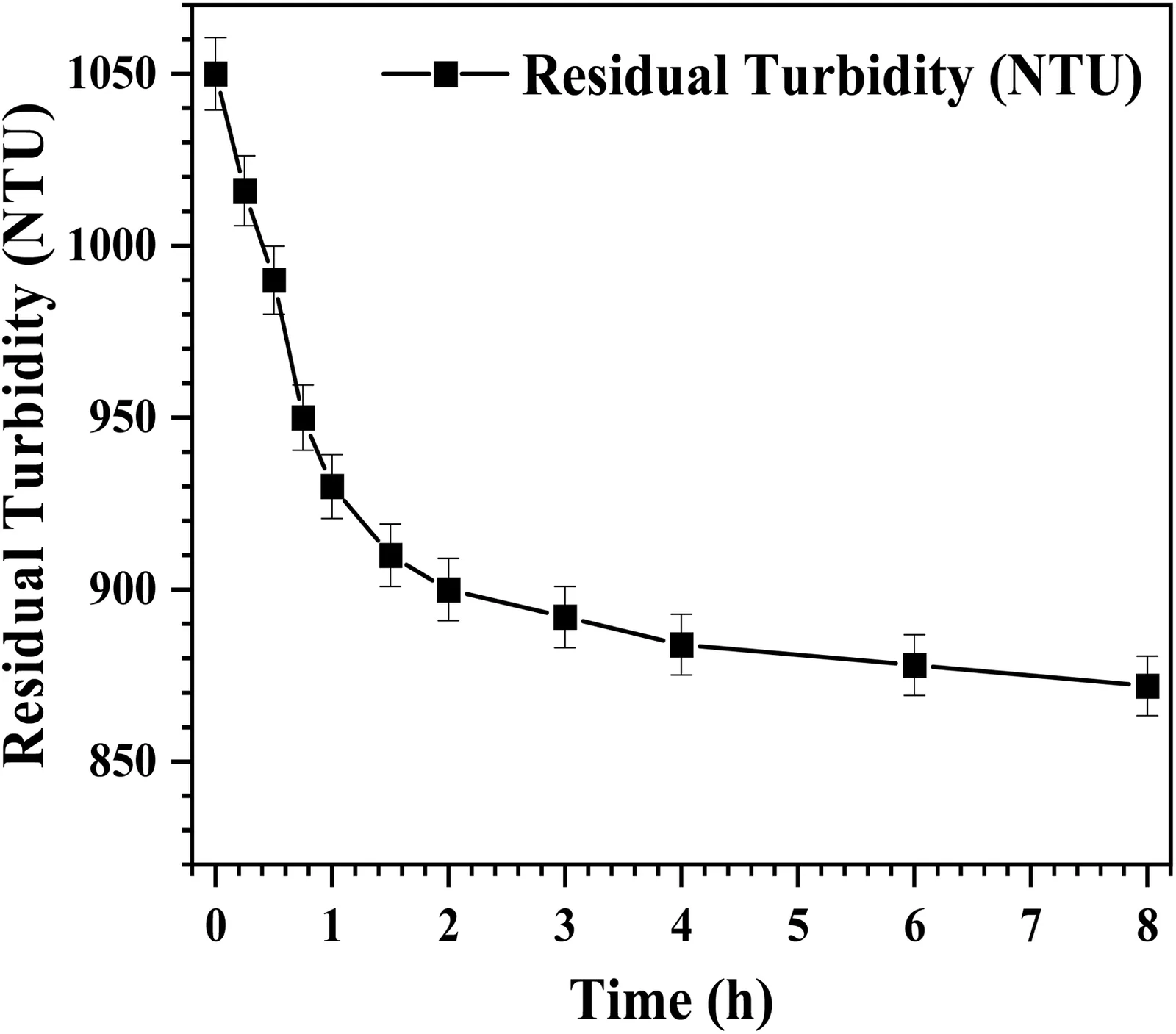
Effect of settling time on turbidity removal: residual turbidity *vs.* time of 1% soybean o/w emulsion using PAC as a coagulant (initial oil concentration: 9020 mg L^−1^; initial turbidity: 10 000 NTU; PAC dose: 1200 mg L^−1^).

This transition suggests that the majority of destabilizable oil droplets had been incorporated into flocs during the first phase, with subsequent removal primarily governed by gravitational settling and floc consolidation processes.^60^ It has been demonstrated that a quiescent clarification period of 8–9 hours was sufficient to achieve effective turbidity removal under the experimental conditions. This extended time requirement is characteristic of oil–water emulsion systems, where the density difference between phases and the formation of less dense flocs necessitate longer settling periods compared to conventional turbidity removal applications. The prolonged retention time ensures adequate separation of the flocculated oil phase from the clarified water phase, achieving the desired treatment efficiency.^61^

### 3.3 Treatment with alum

Among the various coagulants available, aluminum sulfate (alum, Al_2_(SO_4_)_3_) remains one of the most extensively used inorganic coagulants due to its effectiveness, availability, and relatively low cost.^62^

#### 3.3.1 Effect of pH

Fig. 6 illustrates the relationship between pH and residual turbidity during the treatment of a 1% (v/v) soybean oil-in-water (o/w) emulsion using alum as a coagulant (with an initial turbidity of 10 000 NTU and alum dose of 50 mg L^−1^). The results show that the pH of the o/w emulsion significantly affects the removal of oil and turbidity. Maximum removal efficacy is observed at pH 6.0, where the lowest residual turbidity is achieved. The oil removal was 96% and the turbidity removal was 95.3%. Both oil and turbidity removal decline outside the pH range of 6–8, with suboptimal performance noted at pH values above 8 and below 6. This optimal pH range aligns with the well-established mechanism of alum coagulation, in which the speciation of hydrolyzed aluminum species—primarily the formation of Al(OH)_3_ precipitates and Al_13_ polycations—promotes charge neutralization and sweep flocculation of emulsified oil droplets and suspended particles. At near-neutral pH, amorphous Al(OH)_3_ solubility is minimum, facilitating aggregation and subsequent removal of colloidal and emulsified contaminants. At lower pH, excess protonation hampers the hydrolysis and reduces floc formation, while at higher pH, the formation of soluble aluminate ion (Al(OH)_4_^−^) reduces the efficiency of charge neutralization and adsorption, resulting in reduced removal of oil and turbidity.^63–65^ Gregor *et al.*^66^ reported optimal coagulation and flocculation with alum in the pH range of 6.0–7.0 for the removal of natural organic matter in water treatment, ascribed to the predominance of favorable hydrolyzed aluminum species and stable floc structure within this range. The current study extends this pH optimization to oil–water emulsions, demonstrating that pH 6.0 yields maximal oil and turbidity reduction—consistent with both the theoretical and empirical understanding of alum coagulation. Consequently, subsequent experiments and treatment protocols were standardized at pH 6.0 to ensure maximum removal efficacy.

**Fig. 6:**
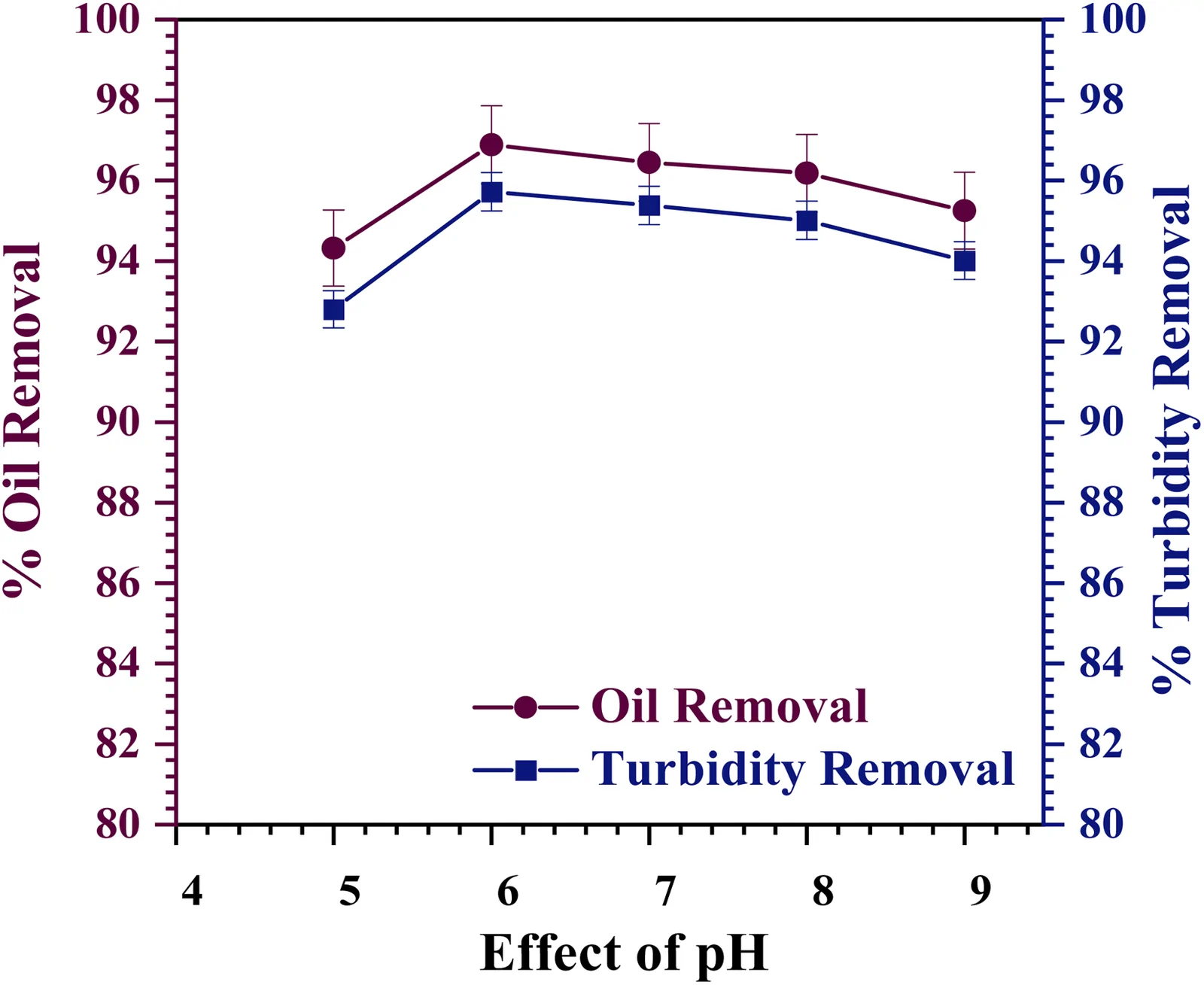
Effect of pH on the removal of oil and turbidity from soybean-oil emulsion using alum as coagulant: residual turbidity *vs.* pH for the 1% soybean o/w emulsion using alum as a coagulant (initial turbidity: 10 000 NTU; alum dose: 50 mg L^−1^).

#### 3.3.2 Effect of dosages of alum on oil removal

The systematic investigation of alum dosage effects on oil and turbidity removal from soybean oil-in-water emulsions, as presented in Fig. 7, demonstrates a clear dose–response relationship across varying oil concentrations (1–4% v/v). The experimental data reveal that increasing alum dosage consistently reduces both residual oil concentration and residual turbidity, indicating enhanced coagulation efficiency with higher coagulant loading. The linear relationship between alum dose and oil concentration is probably valid only within the range of conditions that were tested. This relationship is attributed to improved charge neutralization of emulsified oil droplets and enhanced floc formation kinetics, facilitating more effective phase separation through sedimentation and flotation mechanisms.^67^

**Fig. 7:**
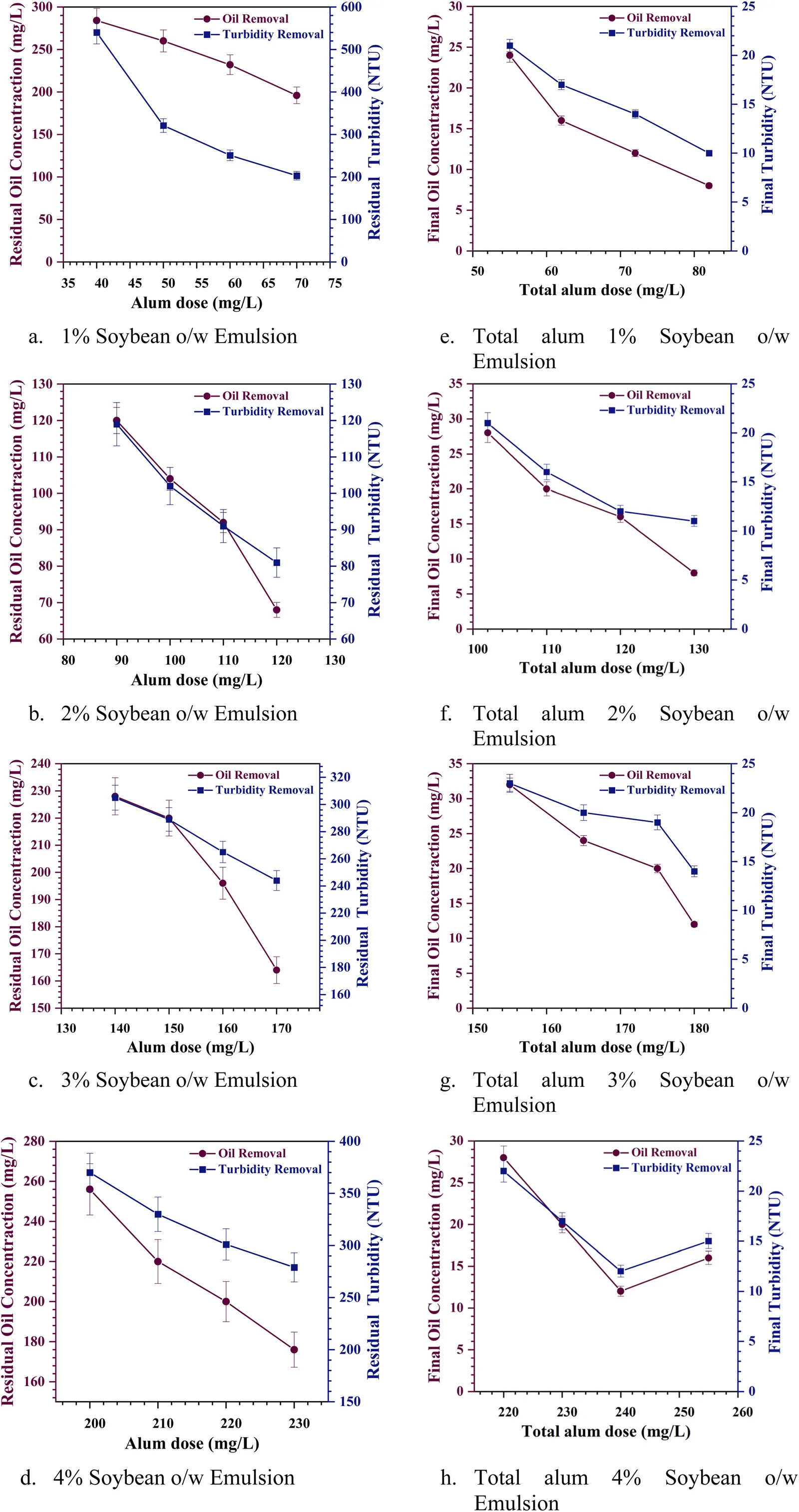
Effect of alum dosage on residual oil concentration and turbidity of soybean oil–water emulsions. (a–d) First-stage alum coagulation of 1%, 2%, 3%, and 4% soybean oil-in-water emulsions, respectively. (e–h) Second-stage treatment of the corresponding supernatants obtained after first-stage coagulation. A fixed additional alum dose of 15 mg L^−1^ was applied to each recovered supernatant. The doses presented in panels (e–h) represent cumulative alum doses, calculated as the respective first-stage alum dose plus 15 mg L^−1^.

However, the inadequacy of single-stage alum treatment to achieve the prescribed discharge limit of 10 mg L^−1^ oil concentration necessitated the implementation of a two-stage coagulation process. The alum dosages used in the two-stage treatment are listed in Table S3. This sequential treatment approach provided the additional removal capacity required to meet regulatory compliance standards. This resulted in achieving oil concentration of 8 mg L^−1^ and 6 mg L^−1^ for 1% and 2% soyabean o/w emulsion, as shown in Fig. 7e and f. The two-stage methodology represents a well-established practice in water treatment engineering, particularly for challenging wastewaters containing high concentrations of emulsified contaminants. The enhanced performance of multi-stage systems is attributed to improved contact time between coagulant and target particles, optimized pH conditions for successive treatment stages, and more complete destabilization of colloidal systems through sequential chemical addition.^57^ The optimal alum dosages determined for two-stage treatment demonstrate a linear correlation with initial oil concentration: 82, 130, 180, and 240 mg L^−1^ for 1%, 2%, 3%, and 4% oil emulsions, respectively (Fig. 7e–h). This proportional relationship indicates a stoichiometric requirement of approximately 60 mg L^−1^ alum per 1% increase in oil concentration, providing a rational basis for process scaling and design optimization. Since the required dosages are consistent and predictable, treatment plants can use automated control systems at industrial scales that automatically adjust in real-time as the incoming oil concentration varies.^68^ However, the approach requires careful consideration of increased chemical consumption costs, elevated sludge production requiring appropriate disposal strategies, and the need for pH conditioning of final effluent. Future research opportunities include investigation of hybrid coagulant systems combining alum with natural coagulants to reduce chemical requirements, process intensification through optimized mixing and contact time parameters, and development of intelligent dosage control systems based on real-time water quality monitoring.^68–70^

#### 3.3.3 Effect of settling time

The time-dependent turbidity removal profile (Fig. 8) demonstrates that following coagulant addition and pH adjustment to 6.0, the emulsion treatment exhibits biphasic kinetics. Turbidity decreased rapidly during the initial stage, achieving approximately 85% removal within the first 180 minutes, followed by a slower removal phase that reached 97% total turbidity reduction at 360 minutes (6 hours), beyond which residual turbidity plateaued at approximately 300 NTU, indicating equilibrium conditions. By the completion of 8 h, the residual oil concentration was 360 mg L^−1^.

**Fig. 8:**
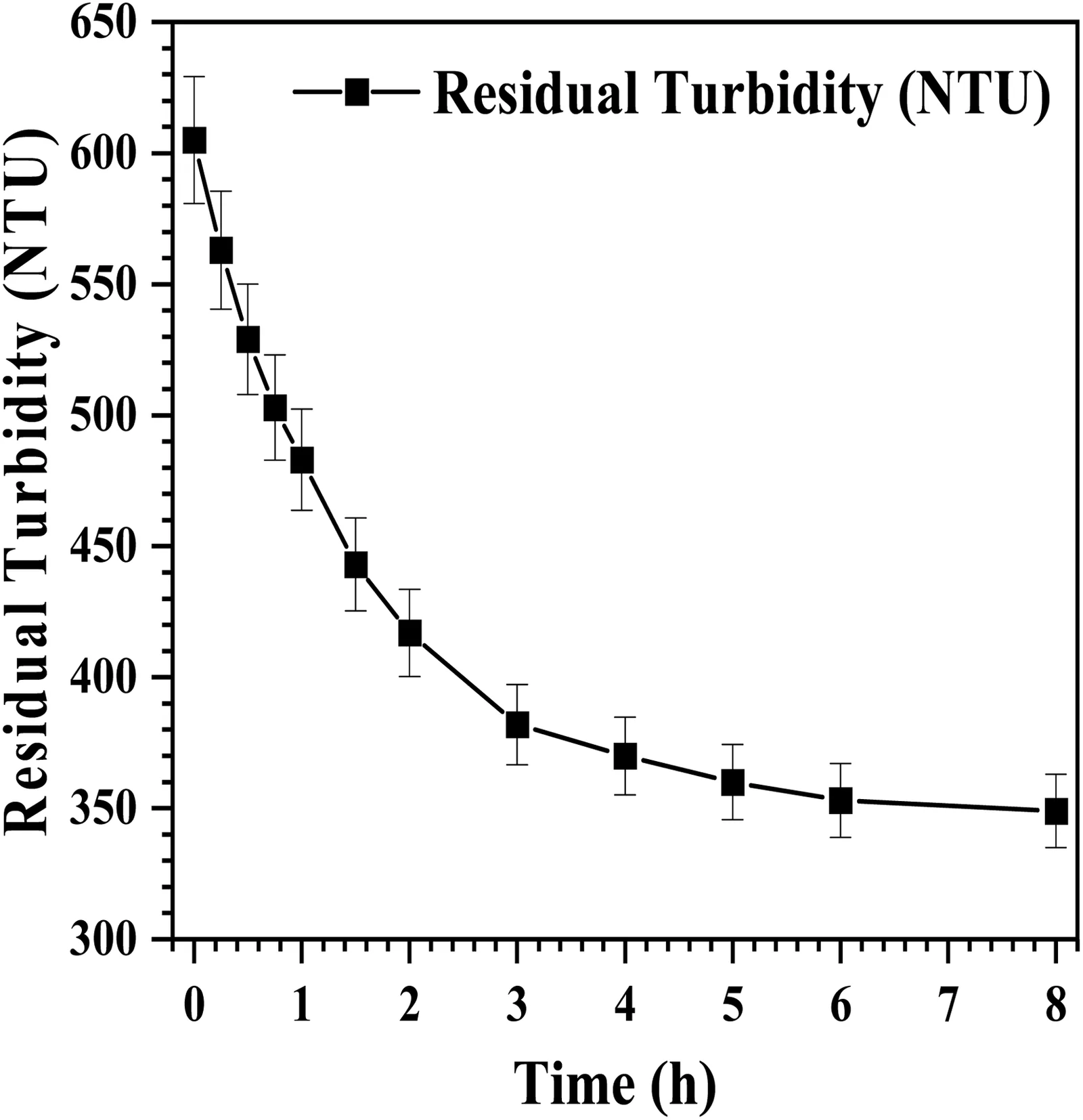
Effect of settling time on turbidity removal: residual turbidity *vs.* time for the 1% soyabean o/w emulsion using alum as a coagulant (initial turbidity: 10 000 NTU; alum dose: 80 mg L^−1^).

In the early stage, rapid collision and aggregation of destabilized oil droplets result in efficient floc formation. Importantly, given the buoyant nature of oil-laden flocs (oil density ∼0.92 g cm^−3^*versus* water at 1.0 g cm^−3^), the primary separation mechanism is flotation rather than gravitational settling. The oil-coagulant flocs rise to the surface, forming a concentrated layer that can be skimmed, while denser inorganic and particulate matter may settle. As the concentration of unaggregated droplets diminishes, the collision frequency decreases, leading to slower turbidity reduction in later stages. The attainment of near-constant residual turbidity at 360 minutes confirms completion of bulk flocculation and highlights the sufficiency of this retention time for practical treatment applications. This emphasizes the importance of optimizing both chemical parameters (such as pH and coagulant dose) and hydraulic factors (mixing intensity, contact time, and flotation/settling periods) to achieve efficient, cost-effective removal of emulsified oil and turbidity in wastewater treatment processes.^71,72^

It should be noted that the oil removal and turbidity removal percentages reported in this study are not expected to be numerically equivalent. The gravimetric oil measurement is proportional to the total mass of dispersed oil, whereas nephelometric turbidity is proportional to the light-scattering intensity of the suspension, which depends nonlinearly on particle size distribution, number concentration, and refractive index contrast (Mie scattering). Because coagulation preferentially removes larger droplets, the residual population is enriched in submicron droplets that scatter light strongly relative to their mass fraction. In addition, for PAC and alum, residual aluminum hydroxide precipitates and flocs contribute to turbidity but not to the measured oil concentration. Turbidity is therefore used in this study as a relative operational indicator of clarification, while all quantitative oil values are based on the gravimetric BIS extraction method.^73,74^

## 4. Conclusion

A comparative assessment of HCl-induced acidification and the coagulation treatments using PAC and alum for concentrated soybean oil-in-water emulsions demonstrates differences in both removal efficiencies and underlying treatment mechanisms. HCl acts principally through interfacial film disruption and charge neutralization, rapidly destabilizing emulsions but leaving a fraction of submicron oil droplets resistant to removal, thus limiting its performance against stringent discharge criteria. PAC removes oil and turbidity efficiently within a narrow optimal pH and dose range, operating mainly *via* polymeric bridging and sweep flocculation—processes that enhance aggregation and facilitate the separation of emulsified oil when pH and dosage are carefully matched to emulsion conditions. Alum, by contrast, relies on hydrolysis-mediated sweep flocculation characterized by the formation of voluminous precipitates that enmesh oil droplets, but with generally slower kinetics; only a multi-stage alum process provides both the removal efficiency and compliance with regulatory oil discharge limits observed in this study. While alum achieves the highest oil (>80%) and turbidity (>96%) removal rates (Fig. 7) in a two-stage process, the increased chemical consumption and sludge generation highlight the practical limitations of conventional coagulation for high-strength wastewater. Thus, alum-based multistage coagulation should be regarded as an effective treatment option for the studied emulsion, rather than a universally optimal solution. The practical viability of this process depends on wastewater-specific optimization. Moving forward, innovations such as hybrid coagulants, process intensification, and smart control strategies will be pivotal in overcoming these limitations, advancing both the efficiency and sustainability of oily wastewater remediation technologies.

## Conflicts of interest

There are no conflicts to declare.

## Supplementary Material

RA-016-D6RA05919H-s001

## Data Availability

The data supporting this study are available in the article and its supplementary information (SI). Supplementary information: Tables S1–S3 summarize the experimental conditions, treatment outcomes, and PAC and alum dosing strategies for the demulsification and coagulation of 1–4% (v/v) soybean oil-in-water emulsions. See DOI: https://doi.org/10.1039/d6ra05919h.
