# Supplementary material for: Acid-induced demulsification versus alum and polyaluminium chloride coagulation for oil and turbidity removal from concentrated soybean oil-in-water emulsions
Source: RSC Adv. 2026 Oct 21. Online ahead of print. doi: 10.1039/d6ra05919h (PMC13685709; doi:10.1039/d6ra05919h)
Supplement: Supplementary file 1 [file RA-OLF-D6RA05919H-s001.pdf]

Supplementary Information (SI) for RSC Advances.
This journal is © The Royal Society of Chemistry

Supplementary Information

Acid-Induced Demulsification versus Alum and Polyaluminium Chloride Coagulation for Oil and Turbidity Removal from Concentrated Soybean Oil-in-Water Emulsions

Praveen Kumar[a*], Shilpi Verma[b], Amit Verma[c], Vimal Chandra Srivastava[c]

[a]Faculty of Chemistry and Chemical Technology, University of Ljubljana, 1000 Ljubljana, Slovenia.

[b]Department of Energy & Environment, Thapar Institute of Engineering & Technology, Patiala-147004, Punjab, India.

[c]Department of Chemical Engineering, Indian Institute of Technology, Roorkee 247667, India

*Corresponding Author: Faculty of Chemistry and Chemical Technology, University of Ljubljana, Ljubljana, 1000, Slovenia

Email: praveen@engineering.ucsb.edu; Kumar.Praveen@fkkt.uni-lj.si

Table S1. Summary of experimental conditions and treatment outcomes for the demulsification and coagulation of 1–4% (v/v) soybean oil-in-water emulsions.

Coagulant	Experimental series	Emulsions tested (v/v)	Optimum pH	Optimum dose (mg/L)	Settling time	Principal outcome
HCl	Dose optimization, single stage	1 - 4%	native	737.5 (1%); per Fig. 1 (2-4%)	30 min	Residual oil 115 - 128 mg/L; above discharge limit

HCl	Second-stage treatment	1 - 4%	native	optimized	30 min	No significant further oil reduction; residual droplets resist acid destabilization
HCl	Settling-time series	1 %	native	737.5	8 h	Turbidity reduced from 10,000 to 290 NTU (97.1% removal)
PAC	pH optimization	1 %	7	1,200	30 min	91.6 % turbidity and oil removal; minimum turbidity 8 - 10 NTU at pH 6.0 - 7.5
PAC	Dose optimization, single stage	1 – 4 %	7	1,300 - 2,350 (increasing with oil load)	30 min	Bell-shaped dose response; re-stabilization beyond optimum
PAC	Two-stage treatment	1 – 4 %	7	optimized per stage	30 min/stage	Residual oil remained above 10 mg/L at all oil loadings
PAC	Settling-time series	1 %	7	1,200	8 - 9 h	Biphasic clarification; transition at ~90 min; flotation-dominated
Alum	pH optimization	1%	6	50	30 min	96 % oil removal; 95.3 % turbidity removal

Alum	Dose optimization, single stage	1 – 4 %	6	per Fig. 7a - d	30 min	Removal increased with dose; residual oil above 10 mg/L
Alum	Two-stage treatment	1 % and 2 %	6	82 and 130 (total)	30 min/stage	Residual oil 8 mg/L (1 %) and 6 mg/L (2 %); compliant with 10 mg/L limit
Alum	Two-stage treatment	3 % and 4 %	6	180 and 240 (total)	30 min/stage	Total dose scales linearly (∼ 60 mg/ L per 1 % oil)
Alum	Settling-time series	1 %	6	80	6 h	97 % turbidity removal; plateau at ∼300 NTU

Table S2: First-stage PAC dose, cumulative PAC dose, and incremental PAC dose applied during second-stage treatment of supernatants from soybean oil-in-water emulsions.

Soybean oil-in-water emulsion	First-stage PAC dose in panels a–d (mg/L)	Cumulative PAC dose in panels e–h (mg/L)	PAC added to supernatant in Stage 2 (mg/L)
1%	1200, 1250, 1300, 1350	1400, 1450, 1500, 1550	200
2%	1600, 1650, 1700, 1750	1800, 1850, 1900, 1950	200
3%	2000, 2050, 2100, 2150	2200, 2250, 2300, 2350	200
4%	2200, 2250, 2300, 2350	2400, 2450, 2500, 2550	200

Table S3: First-stage alum dose, cumulative alum dose, and incremental alum dose applied during second-stage treatment of supernatants from soybean oil-in-water emulsions

Soybean oil-in-water emulsion	First-stage alum dose in panels a–d (mg/L)	Cumulative total alum dose in second-stage panels e–h (mg/L)	Alum added to supernatant in Stage 2 (mg/L)
1%	40, 50, 60, 70	55, 65, 75, 85	15
2%	90, 100, 110, 120	105, 115, 125, 135	15
3%	140, 150, 160, 170	155, 165, 175, 185	15
4%	200, 210, 220, 230	215, 225, 235, 245	15
